# Hepatitis E Virus Seroprevalence among Blood Donors in Southwest Switzerland

**DOI:** 10.1371/journal.pone.0021150

**Published:** 2011-06-20

**Authors:** Annatina Kaufmann, Alain Kenfak-Foguena, Cyril André, Giorgia Canellini, Philippe Bürgisser, Darius Moradpour, Katharine E. A. Darling, Matthias Cavassini

**Affiliations:** 1 Faculty of Biology and Medicine, University of Lausanne, Lausanne, Switzerland; 2 Infectious Diseases Service, Centre Hospitalier Universitaire Vaudois, Lausanne, Switzerland; 3 Service of Immunology and Allergy, Centre Hospitalier Universitaire Vaudois, Lausanne, Switzerland; 4 Service Régional Vaudois de Transfusion Sanguine, Epalinges, Switzerland; 5 Division of Gastroenterology and Hepatology, Centre Hospitalier Universitaire Vaudois, Lausanne, Switzerland; Saint Louis University, United States of America

## Abstract

**Aim:**

The aim of this study was to determine the seroprevalence of Hepatitis E virus (HEV) among blood donors in southwest Switzerland.

**Background:**

HEV is recognized as a food-borne disease in industrialized countries, transmitted mainly through pork meat. Cases of transmission through blood transfusion have also been reported. Recent studies have revealed seroprevalence rates of 13.5%, 16.6% and 20.6% among blood donors in England, France and Denmark, respectively.

**Methods:**

We analyzed 550 consecutive blood donor samples collected in the region of Lausanne, canton of Vaud, Switzerland, for the presence of anti-HEV IgG, using the MP Diagnostics HEV ELISA kit. For each donor, we documented age, sex and alanine aminotransferase (ALT) value.

**Results:**

The study panel was composed of 332 men (60.4%) and 218 women (39.6%). Overall, anti-HEV IgG was found in 27 of 550 samples (4.9%). The seroprevalence was 5.4% (18/332) in men and 4.1% (9/218) in women. The presence of anti-HEV IgG was not correlated with age, gender or ALT values. However, we observed a peak in seroprevalence of 5.3% in individuals aged 51 to 70 years old.

**Conclusions:**

Compared with other European countries, HEV seroprevalence among blood donors in southwest Switzerland is low. The low seroprevalence may be explained by the sensitivity of commercial tests used and/or the strict regulation of animal and meat imports. Data regarding HEV prevalence in Swiss livestock are lacking and merit exploration.

## Introduction

Hepatitis E virus (HEV) was discovered in 1983 and cloned in 1991. The virus is transmitted predominantly by way of the enteral route and may cause waterborne epidemics of hepatitis in developing countries. Whereas human-to-human transmission appears to be exceptional, mother-to-child and blood transfusion transmission have been described [1], [2].

HEV isolates are classified into five major genotypes which belong to the same serotype. Genotypes differ with respect to host species and epidemiological distribution. Genotypes 1 and 2 infect only humans and are endemic in many parts of Asia, Africa and South America. Genotypes 3 and 4 infect humans, pigs and other animal species. Genotype 3 causes sporadic cases of acute hepatitis in North and South America, Europe and Asia whereas genotype 4 is essentially restricted to Asia. Genotype 5 infects avian species [2], [3].

Imported and autochthonous cases (mainly genotype 3) are observed in developed countries. For reasons that are incompletely understood, high HEV seroprevalence rates have been found among blood donors in southwest France (16.6%), southwest England (16%), Denmark (20.6%) and the USA (18.3%) (Table 1), with a large proportion of infections acquired locally [4]–[8].

**Table 1:** Comparison of HEV seroprevalence in developed countries.

Refe-rence	Country of study	Seropre-valence %	Year of publication	Subjects studied (n)	Sex ratio m/f	Median Age	Laboratory test used
[23]	Netherlands	1.1	1993	1275	*na*	*na*	Abbott, Diagnostics Biotechnology
[18]	Italy	1.0	1994	948	2.2	36.9	Abbott
[15]	Switzerland	3.2	1994	94	*na*	*na*	Abbott, confirmation by Western-blot assay
[24]	Spain	2.8	1998	863	*na*	*na*	Abbott, confirmation by Western-blot assay
[25]	NW Greece	0.2	1998	2636	5.38	42	Abbott
[25]	W Greece	0.5	1998	280	1.9	41	Abbott
[8]	USA	18.3	2002	400	*na*	*na*	In-house
[17]	N France	3.2	2007	1998	1.5	*a*	Genelabs Diagnostics
[4]	Denmark	20.6	2008	461	*na*	*na*	In-house
[5]	England	16	2008	500	*na*	*na*	Wantai
[7]	SW France	16.6	2008	529	1.74	41	Genelabs Diagnostics
[26]	SW England	15.8	2008	487	*na*	*na*	Wantai
This Study	Switzerland	4.9	2010	550	1.52	55	MP Biomedicals, formerly Genelabs Diagnostics

Abbreviations: *na*, data not available.

Attempts to explain these findings led to the discovery of new transmission routes, including zoonotic sources. Some genotypes, namely 3 and 4, are able to infect pigs, wild boars and other mammals. Viral RNA has been found in pig livers sold commercially [9]. Pork and other meat products, particularly if undercooked, are thus considered an important transmission route in developed countries [1]. Risk factors for HEV infection include professional exposure to animals (farmers, hunters), age (older populations) and male gender. Other groups considered at high risk for HEV infection are hemodialysis patients, injecting drug users and prisoners [10].

HEV infection is often asymptomatic but can induce a self-limited acute hepatitis, similar to hepatitis A. In pregnant women, HEV severity is increased with mortality rates up to 20%. HEV infection can also be severe in individuals with underlying liver disease in whom mortality rate may reach up to 60% [2], [11]. Cases of chronic HEV infection have been documented in immunosuppressed patients (solid organ transplant recipients, patients treated for malignancies or those infected with HIV [12]–[14].

There are no recent data regarding HEV seroprevalence in Switzerland. In 1994, Lavanchy *et al* reported a seroprevalence of 3.2% among 94 blood donors [15]. In the light of the recent high HEV seroprevalence reported in various European countries, we aimed to assess the current HEV seroprevalence rate among blood donors in Switzerland.

## Materials and Methods

### Ethics statement

All donors had previously completed the national medical questionnaire to verify that they fulfilled the criteria for blood donation and all had provided written consent for the use of blood samples in medical research after anonymization. The study was approved by the Ethical Committee of the Canton of Vaud, Switzerland.

### Population and sample collection

Anonymized blood samples were collected consecutively in November 2009, from 550 blood donors living in the region of Lausanne, canton of Vaud, in southwest Switzerland. This region comprises both urban and rural areas, the latter being used for both arable and livestock-based farming. In order to study a population close to the general population, blood collections at university campuses and army centers were excluded.

### Anti-HEV serology

**Figure 1:**
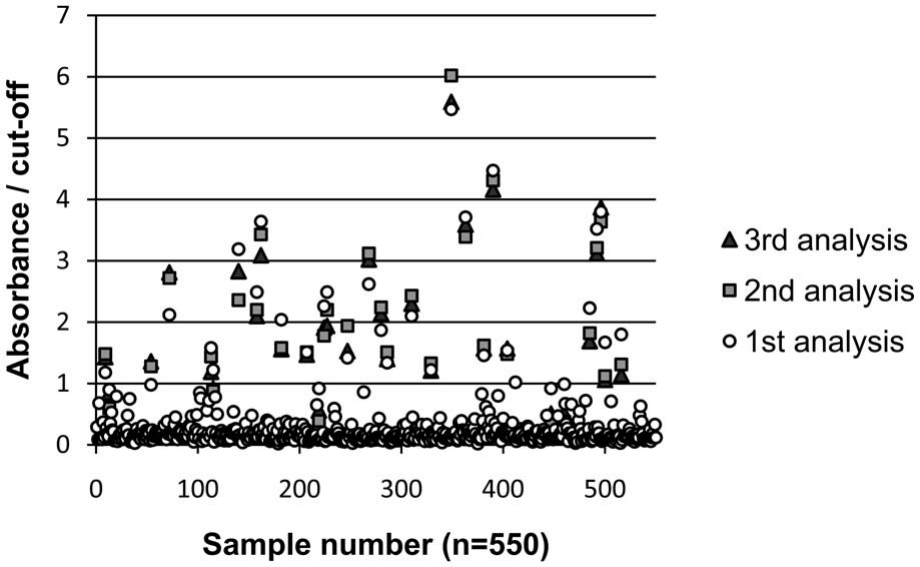
Absorbance/cut-off ratios observed. Samples with initial absorbance/cut-off ratio value>0.90 were retested twice (analyses 2 and 3) and considered positive if both the 2^nd^ and 3^rd^ ratio values were ≥1.0.

Serum anti-HEV IgG was detected by the MP Diagnostics HEV ELISA kit (MP Biomedicals, formerly Genelabs Diagnostics, Singapore). This assay uses three recombinant polypeptides derived from the 3′ termini of open reading frame (ORF) 2 (42 amino acids) and ORF3 (33 amino acids) from Burmese and Mexican prototype sequences (genotypes 1 and 2, respectively). The tests were performed according to the manufacturer's instructions. Initially reactive samples were retested and those with a repeatedly reactive result were considered positive. The absorbance/cut-off ratios observed for all samples are depicted in Figure 1. According to the manufacturer, kit sensitivity and specificity are 97% and 98%, respectively, in regions of low endemicity [16].

### Data collection

For each blood donation we obtained the results of the routine screening tests performed by the Blood Transfusion Center, namely anti-HIV and HIV PCR, HBsAg and HBV PCR, anti-HCV and HCV PCR, TPHA and alanine aminotransferase (ALT). Age and gender for each donor were also collected from the blood bank database.

### Statistical analyses

The Lorenz formula was used for sample size calculation, aiming for a degree of precision of 1.5% and a confidence interval of 95%. Assuming an anti-HEV prevalence of 3%, we estimated a minimal sample size requirement of 500 individuals [15].

Data analysis included descriptive statistics of means (with standard deviation) and medians (with 90% range), depending on the distribution of the data. Categorical data were assessed in two-way contingency table analyses using Pearson Χ2 tests or Fisher's exact test when sample size was small. Odds ratios with 95% confidence intervals were calculated.

All statistical analysis was performed using SAS 9.1 (SAS Institute Inc., Cary, NC) where *P*<0.05 was considered significant.

## Results

General characteristics of the 550 blood donors examined are summarized in Table 2. The study panel was composed of 332 men and 218 women. Their age ranged from 27 to 86 years with a median of 55 years (IQR 46–63 years). 193 donors (35.1%) were aged 50 years or less, while 357 (64.9%) were older than 50 years. Median ALT value was 18 IU/l (IQR 14–25 IU/l), the highest being 84 IU/l. Only 4 donors (0.73%) had ALT values over 60 IU/L (normal range, 11–60 IU/L).

**Table 2:** Characteristics of the study population.

		All donors	HEV IgG negative	HEV IgG positive
		n = 550	n = 523 (95.1%)	n = 27 (4.9%)
Sex	Male	332	314 (94.6%)	18 (5.4%)
	Female	218	209 (95.9%)	9 (4.1%)
ALT	≤60 IU/L	546	519 (95.1%)	27 (4.9%)
	61–100 IU/L	4	4 (100%)	0 (0%)
Age	18–30	6	6 .0 (100%)	0 (0%)
	31–50	187	178 (95.2%)	9 (4.8%)
	51–70	320	303 (94.7%)	17 (5.3%)
	>70	37	36 (97.3%)	1 (2.7%)

Abbreviations: HEV, hepatitis E virus; ALT, alanine aminotransferase.

Overall, 27/550 blood samples (4.9%) tested positive for IgG anti-HEV and included 18 samples from men (5.4%) and 9 from women (4.1%) (*P* = 0.5). HEV seroprevalence varied with age with a peak at 5.3% (17/303) among donors aged 51 to 70 years old (*P* = 0.9) (Table 2). Only 6 donors aged 30 years old and younger were tested, none of whom had a positive result. None of the samples which tested positive for anti-HEV were associated with ALT elevation.

None of the blood samples tested positive for anti-HIV, HIV RNA, HBsAg, HCV RNA or TPHA. One sample (0.18%) tested positive for anti-HCV but negative for anti-HEV antibodies.

## Discussion

We screened 550 blood samples from blood donors in southwest Switzerland and found that HEV seroprevalence was 4.9%. This seroprevalence rate is considerably lower than that reported in blood donors in southwest France, southwest England, Denmark in 2008 and the USA in 2002 [4], [5], [7], [8]. However, our rate was similar to that reported in Northern France (3.2%) in 2007 and in Italy (0.95%) in 1994 [17], [18]. Comparisons between studies are difficult due to differences in the demographics of the population studied and in the HEV antibody detection assays used. The various commercially available tests show important differences in sensitivity. As recently published by Bendall *et al*, the MP Diagnostics HEV ELISA kit that we used may underestimate HEV seroprevalence when compared to the assay from Wantai (Beijing, China) [19]. In their study, Bendall *et al* compared both methods by testing 500 blood samples from blood donors in Bristol, UK, and found a seroprevalence of 3.6% using the MP Diagnostics kit and 16.2% using the Wantai assay. Sensitivity and specificity of laboratory tests might further depend upon prevalence, as well as on the viral genotype present in the study population. Thus, an HEV ELISA based on genotypes 1 and 2 might not completely represent genotypes 3 and 4 present in developed countries. In the absence of standardized commercially available confirmatory assays, such as Western blots, differences in seroprevalence rates between different populations must be interpreted with caution.

Our figure of 4.9% is comparable to that observed among blood donors by Lavanchy *et al* in Switzerland in 1994 (3.2%) [15]. The Lavanchy study used an Abbott HEV ELISA kit with a Western blot confirmation test. As this assay is not the same as the MP Diagnostics HEV ELISA kit used in our study, we cannot state with certainty that HEV prevalence in Switzerland has been stable since 1994. In England, Ijaz conducted a retrospective screening study comparing seroprevalence rates in blood samples taken in 1991 and 2004 and reported similar seroprevalence rates among the two groups (13.0% in 1991 and 13.5% in 2004) [20].

Age is known to be correlated with higher HEV seroprevalence rates. In the study cited above, Ijaz identified as a risk factor the fact of having lived between 1950 and 1959, rather than being of increased age *per se* [20]. Other studies have found older age to be a risk factor for anti-HEV positivity [4], [8]. In our study, older donors tended to have higher HEV seroprevalence rates, although differences were not statistically significant (Table 2).

Blood donors are generally healthy, without any recent history of hepatitis or other illness, or of travel to regions where viral hepatitis is endemic. Studies based on the general population or other specific subgroups report results comparable to blood donors. In the USA, a seroprevalence rate of 18.3% was observed among blood donors and 21.0% in the general population [8], [21]. In the USA, there are major differences between different parts of the country, which may be accounted for, at least in part, by different habits of pork meat consumption [8]. In England, a seroprevalence rate of 16% was reported among blood donors and of 13.5% in the general population [5], [20]. In a recent Swiss study of more than 700 HIV-infected patients with unexplained elevated ALT values, only 2.6% tested positive for anti-HEV IgG using the MP Diagnostics HEV ELISA Kit, supporting the observation of a relatively low HEV seroprevalence in Switzerland [13].

As HEV infection in developed countries seems to be a zoonotic disease transmitted primarily through contaminated meat, we suspect HEV prevalence to be low in pigs and other mammals in Switzerland. Over 90% of the pork meat consumed in the country is produced by regional farmers (Swiss Federal Statistical Office http://www.bfs.admin.ch//bfs/portal/fr/index.html). However, the current situation of HEV prevalence among pigs and other mammals in Switzerland remains unknown. Another possible reason for the lower seroprevalence we observed is that the distribution of risk factors such as profession, hobbies, diet, social status, religion or origin may differ between Swiss blood donors and those in other countries. We were unable to assess these factors in detail in our study population. Further studies are required to clarify the epidemiology and risk factors for HEV infection in Switzerland and indeed in other countries.

As the presence of HEV RNA was not examined in our study, we cannot draw conclusions from our results regarding active HEV prevalence in blood donors and the risk of HEV transmission by blood transfusion. Further studies are needed to assess the potential benefit of adding HEV RNA screening of blood products to the current blood donor selection criteria. Exploring the epidemiology and risk factors for HEV infection may result in implications regarding the safety of blood transfusions in the future. Screening based on ALT level is unsatisfactory as ALT elevation, when it occurs, does so after the onset of symptoms by which time the HEV RNA peak has usually subsided [22]. We need to wait until HEV PCR is commercially available as a screening tool before we can provide more data in this area.

In summary, we have found an HEV seroprevalence of 4.9% among blood donors in southwest Switzerland, using the MP Diagnostics HEV ELISA kit. Our low seroprevalence may be related to the commercial test we used or to the prevalence of HEV among Swiss livestock. As both the choice of diagnostic test and HEV epidemiology may have implications for public health in general, and blood product screening in particular, these topics merit further study.
